# Some Symptoms of Sycosis

**Published:** 1888-08

**Authors:** George H. Clark

**Affiliations:** Germantown, Philada.


### SOME SYMPTOMS OF SYCOSIS.

George H. Clark, M. D., Germantown, Philada.

(Read before the International Hahnemannian Association at Niagara Falls, June, 1888.)

While the Hahnemannian is often taunted because of a presumed lack of knowledge of pathology, it can easily be demonstrated that he is the only one whose teachings are such as to give him a correct idea of the value of the various pathological states. And this can be proved by his every-day experience. If the physician who is in the habit of suppressing disease manifestations by internal drugging, and external applications had sufficient acuity of vision, so as to be enabled to see beyond the immediate present, he would be better able to apply beneficially what he has been taught respecting various affections—provided that teaching be supplemented by a knowledge of Hahnemann's three principal miasmata. In no condition is this more evident than in sycosis. The term sycosis, in the vocabulary of the allopath, is confined to "a folliculitis affecting the hair follicles of the face, particularly those of the chin and whiskers." If the follower of Hahnemann were able to narrow the definition of this term to a simple folliculitis, it would be all the better for mankind.

To him it means a state of the system that may be described as horrible.

Enabled as he is by his real knowledge of pathology to estimate at their true value the various symptoms of disease—particularly the lamentable conditions due to suppressed diseases—his regard for human welfare always calls forth his sympathy for the victim of the terrible affection he knows as sycosis. In the majority of cases, where it is not hereditary, its worst forms are due to maltreatment, and consequent suppression of the sycotic form of gonorrhœa. There are no means by which the primary gonorrhœa can be classified as sycotic or non-sycotic; hence the importance of avoiding any form of treatment that will suppress.

There is less danger (indeed, there is none) in permitting the discharge to continue, even for an indefinite period, than in resorting to injections.

As insidious in its approach as many other scourges, sycosis may be latent for a long time. Sooner or later it will become manifest, and then, if one be familiar with the character of the various ailments which arise, he will readily recognize the importance he should attach to warning all who come to him with any of its primary forms of the suffering and misery that are inevitable unless proper treatment be applied. And that proper treatment is Hahnemannian Homœopathy alone. Like syphilis, it attacks any and every organ and tissue, and in many instances its effects are even worse.

Its most unvarying symptom is *great anxiety*. This anxiety is not paroxysmal, but constant, and the victim of sycosis is always in this pitiable condition.

After Hahnemann, Autenreith, and Bœnninghausen, we are indebted to Granvogl for further elaborating our knowledge of sycosis. He has added much to what was known on this subject before his time, and should, therefore, be given due credit.

It was he who pointed out that the so-called leucæmia of Virchow is, beyond doubt, a form of sycosis, and he taught the so-called physiological school the worthlessness of their efforts to cure this malady. It will compensate us to utilize the labors of Granvogl in this field, and for much of what follows I am indebted to his work.

The anxiety already noted is not simply the concern or solicitude respecting some future event, but it is a constant state of restlessness, with a general feeling of indisposition, and a distressing sense of oppression at the epigastrium. It may be accurately described as anguish.

The mental state is such that there is great confusion of memory; in truth, the memory is very much impaired. The victim is afraid to write even a short note, for fear of misspelling, or of so expressing himself as to be misunderstood. It may be summed up by saying there is a constant state of brain-fag—a sense of an inability to apply himself to anything requiring connected thought.

There is an incomprehensible melancholic mood, with lassitude. Even a short walk fatigues him so much, both mentally and physically, that he is unable to account for it, as he appears strong and hearty to all except a Hahnemannian observer, to whom the expression of his features tells of the mental strain and anxiety.

There is dislike for all labor. He feels that he is too weak to accomplish even what requires but a small expenditure of energy. This lassitude is marked, and is a prominent characteristic.

There is great irritability. Even his own children, in whom he formerly took much delight, annoy him by their presence; and yet he does not possess sufficient energy to leave the room in which they play. A prey to all emotions, he is truly to be pitied. He is constantly worrying without reason, and the slightest thing causes annoyance.

Though previous to this condition he may have been mentally brilliant, he is now a mere intellectual wreck.

With all this there is a disposition to suicide. Life is so burdensome that he does not care to live. All the power of which he is capable must be brought to bear to avoid self-destruction.

There is a dull, heavy feeling in the head, particularly in the vertex, with heat and burning in that region, and burning in and about the eyes.

There is a characteristic diarrhœa, worse early in the morning, with much rumbling and rolling in the abdomen. Aphonia is another faithful accompaniment. The above is a correct photograph of three cases which I have had under treatment during the past two years.

If this mental condition is not sufficient to convince any one of the blighting effects of this poison, a description of the various changes found may have that effect.

Granvogl asserts that the profuse greenish mucous secretion in inflammations of the conjunctiva, sclerotica, and cornea, with bright red injection, the gelatinous effusion into the iris and choroidea, are always of sycotic origin, as well as ciliary and facial neuroses from pressure of the bones, especially if they occur in the evening. Trachoma also belongs here. In affections of the hearing apparatus there is deafness due to swelling of the mucous membrane of the eustachian tube, while syphilitic deafness is the result of caries of the bones.

Catarrhal affections in sycosis are found in all the mucous membranes, more often in damp weather and toward spring.

There are also affections commonly named tuberculous by the old school, and affecting the larynx and trachea. While in genuine tuberculous affections burning is complained of, in the sycotic condition simulating tuberculosis there is no burning; but there is a constricting sensation, which is not increased by pressure upon the larynx. The sensation is as though the passage was obstructed by a foreign body, and, as a consequence, there is a constant desire to swallow.

Hoarseness is a prominent symptom, and increases continuously; and there is much dyspnœa, particularly in going upstairs.

One of the patients, above referred to, has had hoarseness very marked for several years, and had an operation performed for what was said to be a growth in the larynx, since which the hoarseness has never left him.

This condition of the respiratory organs usually precedes more deeply seated symptoms, and Granvogl says that there is danger of death from suffocation if the proper treatment is not given. With this there is a hoarse, hard, whistling, barking cough.

Œdema of the glottis belongs to sycosis. "While it frequently occurs only as a swelling, the hoarseness and difficulty of breathing arise partly from ulceration, partly from knotty formations upon the mucous membrane of the trachea, especially upon its glands, or from a change of the vagus or sympathicus, which are found to be surrounded with hard, gland-like swellings, as if by a string of pearls."

A peculiar and striking characteristic is this:

"Those sycotic glandular formations appear even in places where no glands at all are to be found. Scrofulous glandular swellings always have their seat in the glands originally present.

"These formations, varying in size from the head of a pin to enormous dimensions, spread themselves as painless, indolent tumors; while syphilitic glandular swellings, especially of the inguinal glands, are of an inflammatory and painful nature.

"In sycosis these swellings are found in almost all parts of the body, excepting the muscular system—in the omentum, the mesentery, the intestines, the substance of the kidneys, in the testicles, the ovaries, the liver, the spleen, the diaphragm, the lungs, on the pleura, in the substance of the heart, upon the meninges of the brain, on the nerves, and under the epidermis."

Since Granvogl's time we have had nothing added to our knowledge of sycotic asthma. This is his description of it: * * * * * "The sycotic asthma, in consequence of changes which have taken place in the pulmonary tissue, is distinguished from other asthmatic forms by this, that it also, according to subjective feeling, has its seat in the breast, and not, as otherwise, in the larynx ; furthermore, that it occurs paroxysmally and intermittently, and not unfrequently during swelling of the external glands. It disappears spontaneously without leaving a trace." It may also arise in consequence of swellings of the glands at the bifurcation of the trachea, and may be suddenly excited by going up stairs, muscular movement, or mental emotion. Here I wish to say that it is folly to attribute all forms of asthma to sycosis, and it is false teaching to assert that Nat. sul. will cure all cases of sycotic asthma.

Some years ago Dr. Hering told me that his experience with Nat. sul. in sycosis led him to the conclusion that Granvogl had claimed more for that remedy in gonorrhœa and sycosis than it is capable of performing. And he warned me against giving credit to such teaching.

The pneumonia of sycosis is particularly marked by the inexpressible anxiety. Hoarseness and dyspnœa are always present, the latter always out of proportion to the amount of lung involved. It is further characterized by great prostration and copious, greenish, easy expectoration, and an unendurable sensation of weight and tension upon the chest. In this disease emaciation is prominent, and the urine is generally a rose-red color.

Like other forms of pneumonia, if this be improperly treated there follows a condition simulating an often pronounced tuberculosis of the lungs. The pathologists of this day call it chronic interstitial pneumonia. There is induration of tissue, with catarrh, which is followed by obliteration of the bronchi and blood-vessels by fibrinous clots. Granvogl also shows that the thrombosis and embolism of Virchow are forms of sycotic degeneration. And to sycotic origin are to be attributed many forms of tumors, including the fatty, gelatinous, and gummatous, together with some forms of cancer. Skin affections, differing from syphilis in never being coppery-red in color, with a tendency to ulcerate, characterize the exanthemata of sycosis. The ulcers have a foul odor, are jagged on the edges, with a bluish red or dark brown, cracked base, and extend in breadth rather than in depth.

"If not interfered with the ulcers heal from the centre outward," says Granvogl, "and never with any other loss of substance than that which the cicatrix occasions. By its location on the neck, the sternum, the loins, the throat, in the axillæ, on the upper part of the arm, the thigh, the chin, and not unfrequently on the great toe, its course, its quality, and its termination, it never can be confounded with any other ulcer, and all the less since, like all other sycotic forms, it usually heals spontaneously, provided no radical cure by art intervenes, because it forms only transient stages of one and the same, often life-long, process. It heals up, however, only to return with perfect certainty at some other time in some other place, even at the end of years, and that, indeed, as with all sycotic forms, usually during a long period of damp weather or toward spring, provided the process has not gained in extent, and in place of the ulcer another sycotic form has developed itself."

Then the bones may be affected in the chronic form of sycosis, and Granvogl has pointed out the difference between the bone affections of syphilis and sycosis by showing that in sycosis the bones usually affected are those of the nose—though the sycotic ozœna does not possess the fetid odor of the syphilitic form—of the cavity of the mouth, the palate, the upper and lower jaw, the sternum, the sacrum, the ribs, and the spinous processes of the vertebræ. Unlike syphilis, sycosis affects the bones first through the soft parts through the periosteum and without caries. There is "necrosis," with the formation of sequestrum, and "sclerosis." After sycotic necrosis, unlike syphilitic and scrofulous necrosis, there is always formed new bone to repair the loss.

"Rachitis, enchondroma, and pedarthocace" also belong to sycosis.

Bœnninghausen's remarkable cure of the case of volvulus with Thuja was justified from even a pathological standpoint, for there is classed under sycosis not stricture of the urethra only, but also of the œsophagus, the larynx, the trachea, the rectum, and the intestines.

In joint affections in sycosis the joints usually attacked are those of the vertebral column, the lower jaw, and one of the knee and elbow joints. "The pains arise suddenly and at once, with the greatest intensity. Notwithstanding this, no fever sets in, and the integuments remain normal." If they become anchylosed there is persistent enlargement about the joints, a condition similar to arthritis deformans. If all this, added to the mental state, be not sufficient to convince of the power of this sycosis we can enumerate hypertrophy and induration of the testicles, following inflammation, hydrocele, polypi, ischias, trismus, tetanus, and epilepsy.

Truly, a formidable list!

And yet this does not exhaust the complications and combinations which arise in the course of the malady. For further regarding this subject let me recommend you to Hahnemann, to Bœnninghausen, to Lippe, to Granvogl, to Fellger, to Wells, to Bayard, and to all others who have so successfully borne aloft the law of therapeutics as found in the teachings of Hahnemann.

